# Editorial: Implementing Logic Gates in Adoptive Cell Therapy

**DOI:** 10.3389/fimmu.2022.902594

**Published:** 2022-04-29

**Authors:** Gideon Gross, Yaron Carmi, Hinrich Abken

**Affiliations:** ^1^ Laboratory of Immunology, MIGAL - Galilee Research Institute, Kiryat Shmona, Israel; ^2^ Department of Biotechnology, Tel-Hai College, Upper Galilee, Israel; ^3^ Department of Pathology, Sackler Faculty of Medicine, Tel Aviv University, Tel Aviv, Israel; ^4^ Regensburg Center for Interventional Immunology, Department for Genetic Immunotherapy, and University Hospital Regensburg, Regensburg, Germany

**Keywords:** Boolean logic gates, cancer immunotherapy, tumor-associated antigens, tumor microenvironment, precision targeting

Key to success in adoptive cell therapy (ACT) of cancer is distinguishing between target cells to be attacked and innocent cells to be protected. Achieving this goal requires identifying tumor antigens and distinctive characteristics of the tumor microenvironment (TME) that allow unequivocal target recognition and the development of suitable tools for sensing and translating the information into a precise killing program. Non-mutated tumor-associated antigens (TAAs), which are shared by the majority of tumors of a given type and preferably across different types, as well as distinct TME markers, could provide useful keys for target-nontarget discrimination.

The realization that single and strictly tumor-specific cues are generally lacking encouraged efforts to identify tumor-specific combinations of cues, whether present or absent, whose integrated recogtion would allow precision targeting. Following this concept, it became crucial to create new molecular circuits, which implement the principles of Boolean logic gates for guiding targeting specifically towards these combinations. The articles in this Topic address diverse aspects of this challenge, highlighting exciting directions that are currently pursued in this rapidly evolving field.

Most likely, loss of heterozygosity (LOH) creates one of the richest sources of antigen combinations that are targetable by NOT-gated therapy. Owing to massive deletions of chromosomal material that characterize almost all tumors, many of which occurring early at the premalignant stage, descendent tumor cells do not express the protein products of numerous alleles. Among these are extracellular epitopes of cell surface proteins and HLA-I-bound peptides (collectively ‘ligands’) that can be specifically recognized by antigen receptors. When therapeutic T cells express a CAR or TCR (‘activator’) specific to a conventional TAA that is also expressed by a given normal tissue, the co-expression of an inhibitory ligand-specific receptor (iCAR or iTCR), or ‘blocker’, would protect that normal tissue from attack while allowing efficient killing of unprotected tumor cells.

A pioneering study demonstrating the feasibility of an LOH-targeted integrator termed ‘Tmod’ was published in 2020 by A2 Biotherapeutics (1). It was soon followed by a publication from Johns Hopkins University (2), dubbing the corresponding platform ‘NASCAR’, and additional publications from A2 Biotherapeutics (3, 4). In all these works, the NOT module targeted HLA-I products which are frequently lost by many tumors, employing signaling elements derived from LIR-1 (Tmod) and PD-1 (NASCAR) inhibitory receptors.

Here, Manry et al. of the A2 Biotherapeutics group present a comprehensive pharmacological analysis of the functional parameters that govern Tmod, examining iCAR and iTCR models. They show that dominance of blockers over activators, as manifested by reduced activator sensitivity and magnitude of activation is preserved across a surprisingly wide range of antigen ratios. Meanwhile, dominance is more sensitive to changes in blocker versus activator ratios. Nonetheless, it still exhibits sufficient stability over a range that is controllable by genetic engineering. These conclusions provide important guidelines for the future design of NOT-gated circuits targeting a selected pair of antigens offered by the huge landscape of tumor-specific signatures created by LOH.

‘Switch receptors’ allow the translation of an external input into an output of choice by replacing the intracellular signaling portion of an input-sensing receptor by an output-signaling receptor (5, 6). In ACT, switch receptors are used to convert a signaling pathway that is normally initiated upon recognition of a selected ligand at the TME into the preprogrammed pathway, turning, for example, an inhibitory signal into a costimulatory one, e.g. PD1:CD28 switch receptors (7, 8). Along this line, Olguin-Contreras et al. describe a new CD40L:CD28 costimulatory switch receptor, which takes advantage of the presence of the CD40 receptor on TME-resident myeloid cells. After cracking the technical challenge of fusing the ectodomain of a type II transmembrane protein (CD40L) with a type I endodomain (CD28), the authors demonstrate a unique ‘double-strike’ effect. Engagement of the CD40L:CD28 receptor with CD40 on myeloid cells directly boosts the T cell response *via* the CD28 portion and, at the same time, delivers CD40 signaling to the myeloid cells, which could promote their beneficial repolarization in the TME.

TRUCKs (“T cells redirected for unrestricted cytokine mediated killing”) comprise another class of gene circuits, which exploit antigen-mediated activation for recruiting a selected transcription factor (TF) to drive the expression of a transgene that is placed under the control of a TF-responsive promoter (9). Two articles in this collection focus on this strategy. Rudek et al. describe an advanced ‘all-in-one’ retroviral vector, which simultaneously drives the constitutive expression of one gene (here an antitumor CAR) and the activation-induced expression of a second gene (IL-12). The authors demonstrate this approach for the first time in NK cells and show that in contrast to T cells, transgene induction in NK cells required NFκB-binding DNA sequences in the regulatory element. At the same time, a synthetic promoter sequence, which previously outperformed a minimal IL-2 promoter in T cells (10), proved leaky in this system. These findings underscore critical differences between T and NK cells that should be taken into account while designing molecular circuits. In their ‘hypothesis’ article, Khanali et al. propose to couple an AND logic gate to an OR gate with a new TRUCK-like device for targeting, remarkably, four different antigens *via* the products of a single gene cassette. This complex circuit is turned on upon binding of a constitutively expressed chimeric receptor specific to TAA1, which activates the JAK2/STAT4 pathway through the signaling portion of the IL-12Rβ2 subunit. Activation turns on a string of three genes that are tethered by 2A peptides and positioned under the control of a STAT4-responsive promoter: the first and second genes encode anti-TAA2 and anti-TAA3 CARs and the third encodes a bi-specific T-cell engager (BiTE) against the TME-associated fibroblast associated protein (FAP), creating a three-module OR gate.

Finally, the mini review by Savanur et al. focuses on the use of AND and NOT gates in ACT for improving precision of cancer treatment. The authors first examine different types of cues, which are utilized for distinguishing malignant from normal cells, and then review diverse strategies for creating molecular circuits devised to implement these logic gates in the clinical setting.

## Author Contributions

GG made a substantial contribution drafting this Editorial. HA and YC made direct intellectual input, carefully reviewed the draft and approved it for publication. All authors contributed to the article and approved the submitted version.

## Conflict of Interest

The authors declare that the research was conducted in the absence of any commercial or financial relationships that could be construed as a potential conflict of interest.

## Publisher’s Note

All claims expressed in this article are solely those of the authors and do not necessarily represent those of their affiliated organizations, or those of the publisher, the editors and the reviewers. Any product that may be evaluated in this article, or claim that may be made by its manufacturer, is not guaranteed or endorsed by the publisher.
